# Electrical Impedance of Acupuncture Meridians: The Relevance of Subcutaneous Collagenous Bands

**DOI:** 10.1371/journal.pone.0011907

**Published:** 2010-07-30

**Authors:** Andrew C. Ahn, Min Park, Jessica R. Shaw, Claire A. McManus, Ted J. Kaptchuk, Helene M. Langevin

**Affiliations:** 1 Martinos Center for Biomedical Imaging, Massachusetts General Hospital, Charlestown, Massachusetts, United States of America; 2 Division of General Medicine and Primary Care, Beth Israel Deaconess Medical Center, Boston, Massachusetts, United States of America; 3 P-pro Korea Co., Ltd., Seoul, Korea; 4 AltThera Health, Boston, Massachusetts, United States of America; 5 Department of Research and Training, Spaulding Rehabilitation Hospital, Boston, Massachusetts, United States of America; 6 Division for Research and Education in Complementary and Integrative Medical Therapies, Harvard Medical School, Boston, Massachusetts, United States of America; 7 Department of Neurology, University of Vermont, Burlington, Vermont, United States of America; Illinois Institute of Technology, United States of America

## Abstract

**Background:**

The scientific basis for acupuncture meridians is unknown. Past studies have suggested that acupuncture meridians are *physiologically* characterized by low electrical impedance and *anatomically* associated with connective tissue planes. We are interested in seeing whether acupuncture meridians are associated with lower electrical impedance and whether ultrasound-derived measures – specifically echogenic collagenous bands - can account for these impedance differences.

**Methods/Results:**

In 28 healthy subjects, we assessed electrical impedance of skin and underlying subcutaneous connective tissue using a four needle-electrode approach. The impedances were obtained at 10 kHz and 100 kHz frequencies and at three body sites - upper arm (Large Intestine meridian), thigh (Liver), and lower leg (Bladder). Meridian locations were determined by acupuncturists. Ultrasound images were obtained to characterize the anatomical features at each measured site. We found significantly reduced electrical impedance at the Large Intestine meridian compared to adjacent control for both frequencies. No significant decrease in impedance was found at the Liver or Bladder meridian. Greater subcutaneous echogenic densities were significantly associated with reduced impedances in both within-site (meridian vs. adjacent control) and between-site (arm vs. thigh vs. lower leg) analyses. This relationship remained significant in multivariable analyses which also accounted for gender, needle penetration depth, subcutaneous layer thickness, and other ultrasound-derived measures.

**Conclusion/Significance:**

Collagenous bands, represented by increased ultrasound echogenicity, are significantly associated with lower electrical impedance and may account for reduced impedances previously reported at acupuncture meridians. This finding may provide important insights into the nature of acupuncture meridians and the relevance of collagen in bioelectrical measurements.

## Introduction

To this day, the fundamental tenets of acupuncture, the acupuncture point and meridian, remain a mystery. What are they anatomically and how do they function physiologically? Past studies have anatomically linked these Traditional Chinese anatomical structures to neurovascular bundles [1], [2], [3], trigger points [4], [5], [6], and connective tissue fascial planes [7]. Other studies have identified functional correlates including reduced electrical impedance [8], [9], [10] and enhanced migration of nuclear tracers [11], [12], [13], [14], [15], [16]. However, due to multiple study design limitations - including inadequate descriptions of acupuncture point/meridian localization, small sample size, and unexplained statistical analysis - a definitive conclusion regarding the scientific basis of these structures is difficult to establish.

Of all the reported associations, reduced electrical impedance and correlating intermuscular connective tissue planes may be the most compelling. Distinguishing electrical properties have been reported at acupuncture points and meridians since the 1950s and - more than any other functional correlate - have been the subject of inquiry in research studies investigating these traditional Chinese structures. According to these studies, acupuncture points and meridians are characterized by decreased impedance and increased capacitance compared to adjacent controls. The evidence for and against this association was recently reviewed [17]. The anatomical association with intermuscular connective tissue has also been systematically evaluated. Acupuncture meridians and points were found to overlie the fascial planes between muscles, and this association was determined to be statistically significant based on hypergeometric analyses [7]. Heightened mechanical stress arising from needling loose (areolar) connective tissue may further corroborate the hypothesis that connective tissue - particularly, collagen - may mediate biomechanical and other physiological effects of acupuncture [18], [19], [20], [21].

Given these two reported associations, we hypothesized in a previous study that intermuscular connective tissue was the anatomical basis for the reduced electrical impedance reportedly observed at acupuncture meridians. We tested this hypothesis and found that electrical impedance at Pericardium meridian-associated connective tissue was significantly reduced compared to an adjacent muscle control [22]. The Spleen channel segment, on the other hand, showed no statistical difference. This lack of difference was attributed to unintended placement of Spleen-control needles in an adjacent intermuscular plane.

To avoid confounding by an adjacent connective tissue plane and to see whether the findings from the Pericardium (PC) channel were generalizable to other body sites, we assessed the electrical impedance of skin and underlying subcutaneous connective tissue at the Large Intestine (upper arm), Liver (thigh), and Bladder meridians (calf). These sites were originally chosen because they represented a good balance of anatomical locations and meridian types (2 Yang and 1 Yin channel), and the meridians were not located close to another meridian or intermuscular tissue plane. Acupuncturists determined the location of meridian sites, and ultrasound images were obtained at each test site to record any potential structural associations with electrical impedance. There were two primary aims for this study: (1) to determine whether the electrical impedances at acupuncture meridians were significantly lower than impedances at adjacent controls, and (2) to assess whether echogenic collagen was significantly associated with electrical impedance obtained at the test sites.

## Methods

### Ethics Statement

This study was reviewed and approved by the Institutional Review Board at the Beth Israel Deaconess Medical Center. Each study participant read and signed an informed consent form.

### A. Subjects

Twenty eight subjects (19 female, 9 male) were recruited to participate in the study. Participants were recruited via flyers placed throughout Boston campus areas near Beth Israel Deaconess Medical Center and via postings in Craigslist (www.craigslist.org). Subjects were excluded if they were under 18 years old, pregnant, used anticoagulation medications, had history of bleeding disorder, had an implanted ventricular defibrillator, had a chronic skin condition (such as eczema, psoriasis), or had a collagen disorder (scleroderma, mixed connective tissue disorder, Marfan's). To avoid circumstances where needles were too short to penetrate a sufficient proportion of the subcutaneous layer, a BMI less than 30 was also required. Subjects' age was 28.6±7.9 (mean ± SD) years. Demographic representation was: 23 non-Hispanic White, 1 Hispanic, and 4 Asian. Each subject was compensated for participation. The testing was performed in the General Clinical Research Center at the Beth Israel Deaconess Medical Center.

### B. Testing sites

**Figure 1:**
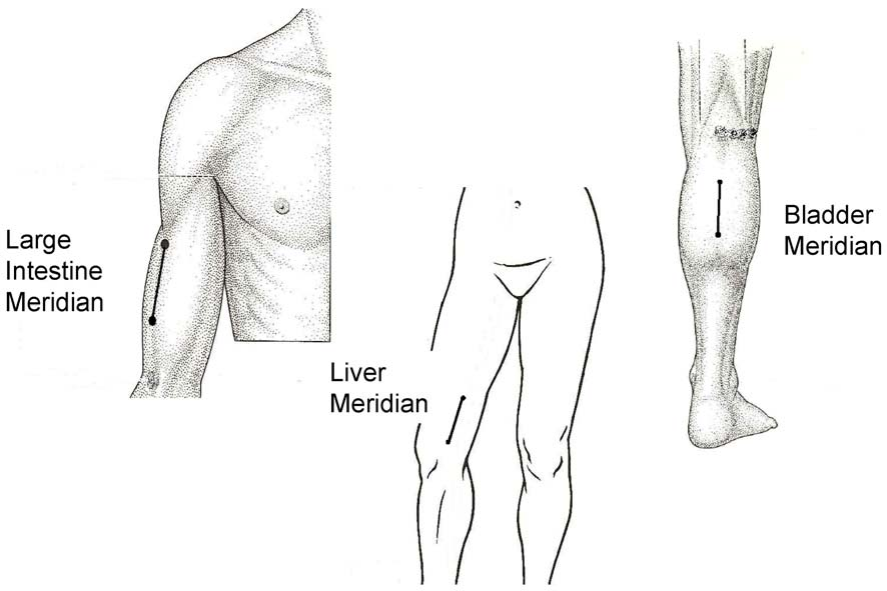
Test Segments for the Three Meridians. The location of the meridian are based on anatomical descriptions given in acupuncture text [24].

Three different segments on the skin surface located along acupuncture meridians were chosen for measurement. These segments were the Large Intestine, Liver and Bladder segments located on the lateral aspect of the upper arm, medial aspect of the thigh and posterior aspect of lower leg respectively (Figure 1).

Large Intestine (LI) segment: The central portion of the LI segment was located at acupoint LI 13 – on the anterolateral aspect of upper arm, 3 cun (a unit of proportional measurements used in acupuncture practice [23]: 1 cun = the width of one's thumb) proximal to LI 11 (lateral end of cubital crease of elbow) in the depression between the lateral border of the biceps and the shaft of the humerus [24]. The control segment for the LI meridian was located 1.3 cm posterior and parallel to the LI segment, overlying the triceps muscle.

Liver segment (LV): The distal portion of LV segment was located on LV 9, between the vastus medialis and Sartorius muscle, 4 cun proximal to LV 8 (just superior to the medial end of the popliteal crease) [24]. The control segment for the LV meridian was located 1.3 cm anterior and parallel to the LV segment, overlying the vastus medialis.

Bladder segment (BL): The distal portion of the BL segment was located on BL 56 – located on the posterior aspect of lower leg, in the center of the belly of the gastrocnemius muscle, 5 cun below BL40 (center of posterior popliteal crease) [24]. The control segment for the BL meridian was located 1.3 cm lateral and parallel to the BL segment, overlying the gastrocnemius.

The meridian segments were identified by two separate acupuncturists and ultimately determined by consensus if any disagreements occurred. The acupuncturists had on average 7 years of experience and each represented a separate acupuncture style – Japanese and traditional Chinese. For both acupuncturists, acupuncture points (as described previously) acted as geographical references for the meridians. Meridian segments were further identified either by areas of tenderness with applied pressure (traditional Chinese) or by qualitative changes in the dampness or temperature of the skin (Japanese).

**Figure 2:**
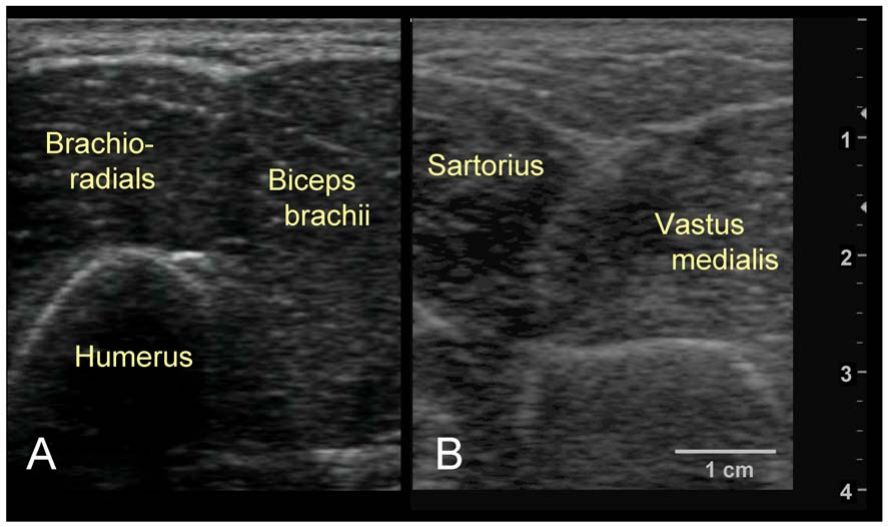
Challenges with Targeting Intermuscular Fascia. (**2A.**) Ultrasound of upper arm. There are frequently no clear distinctive divisions between brachioradialis and biceps brachii muscles as shown in this ultrasound image. (**2B.**) Ultrasound of leg. The fascia between the sartorius and vastus medialis muscles have oblique depth angles.

Although the original intention was to evaluate the intermuscular connective tissue, in our pretest evaluations, the subcutaneous layers in some individuals (even in those with BMI less than 30) were as thick as 4–5 cm in the thigh and upper arm necessitating larger needle penetration depths than what was considered feasible. Moreover, the needle insertion into intermuscular connective tissue planes was complicated by the tight proximity between adjacent muscles (Figure 2). Particularly in the LI and LV channels, the narrow dimensions of the intermuscular space along with oblique angles of the fascial planes made accurate placement of the needles technically challenging. Furthermore, the intermuscular trajectory mapped out by imaging did not consistently match the trajectory mapped out by the acupuncturists. This was particularly true for the intermuscular plane near the LV meridian where the Sartorius muscle runs obliquely along the leg and across the LV meridian path. For these reasons, we decided to focus on the skin and underlying subcutaneous connective tissue in all our subjects. In other words, impedance measurements were limited to the subcutaneous fat region above muscle.

### C. Impedance Measurements

The order of body sites (upper arm vs. thigh vs. lower leg) and channel segment (meridian vs. control) were randomized. When testing the upper arm, subjects were asked to lie supine on a hospital bed with the head elevated at approximately 60 degrees. The right arm was propped over a portable table to expose the lateral aspect of the arm. For testing the thigh, subjects remained supine with the right knee slightly bent and externally rotated in order to have an access to the medial thigh. When testing the calf, the subjects were asked to lie prone and flat to expose the posterior aspect of the left lower leg. If scars, cyst, or infection were present to preclude the accurate testing of electrical impedance, the contralateral extremity was used instead.

A Solartron 1260 impedance gain phase analyzer and a Solartron 1294 biological interface (Solartron Analytical, Hampshire, UK) were used for this study. The combined use of these devices allows for four-electrode measurements to help bypass confounding by electrode polarization. The Solartron 1260 is considered one of the more accurate impedance analyzers available with impedance and phase angle accuracy of 0.1% and 0.1°, respectively, with 1 µV and 1 pA sensitivity, and capabilities of measuring electrical impedance in the frequencies range of 10 µHz to 32 MHz. The 1294 interface has been specifically designed to extend the impedance analyzer to *in-vivo* human settings and is safety compliant and standardized to IEC 601 requirements. The 1260 device used for this particular project were calibrated and standardized by a third-party authority.

After cleaning the skin with alcohol, a sterile adhesive holder (Suture aid booties, Sterion Incorporated, Ham Lake, MN) was placed on the skin at the location. Each holder holds sterile acupuncture needle guide tubes that were each cut to a specified length, depending on the depth of the subcutaneous layer (determined by ultrasound as described below). The needle was inserted to the average depth of the superficial perimuscular fascia (i.e. the fascia overlying the most superficial muscle) at the tested body site. The four meridian guide tubes were placed over the meridian segment, with the proximal guide tube located at the proximal end of the segment. Within meridian and control segments, the guide tubes were 13 mm apart, with the exception of the two inner guide tubes where the interval distance was 25 mm. This creates a scaffold for the needles to maintain consistent (1) inter-needle distance within a given segment, (2) inter-segment distance between meridian and control segments and (3) needle insertion depth. The adhesive holder and needle arrangements have been described and imaged in a previous publication [22].

Four gold-plated needles (Viva 0.25 mm diameter, 40 mm length, Helio Medical Supplies, Inc., San Jose, CA) were connected to the impedance meter device via electrical cables (Teca, Oxford Instruments Medical, Hawthorne, NY). The gold plated acupuncture needles acted as electrodes. The needle was chosen for its metallic handle and its absence of an insulating, silicon layer. The handle of each acupuncture needle was inserted 8 mm into the female end of the connector cable, while the male end was connected to cables connected to the impedance device. Four needles were inserted through each of the four guide tubes overlying either a meridian or control segment and then into the skin and underlying tissues. Each needle was inserted until the hub of the connector contact the end of the guide tube.

A constant 10 µA AC current was introduced at seven separate frequencies ranging from 1 Hz to 1 MHz to the outer two needles while the voltage was measured between the inner two needles. The impedance magnitude and phase angle were stored using either SMART software (Solartron Analytical, Hampshire, UK) or G-plot program (Scribner Associates, Southern Pines, NC). The impedance measures were derived with an embedded autointegration program that calculated the averaged impedance and phase at each frequency. Each frequency sweep took approximately 4–5 minutes. Impedance measurements were made twice at each segment. Other than being 1.3 cm apart, the conditions for measurements at the control and meridian segments were identical.

### D. Ultrasound Images

Before measuring impedance, ultrasound images were obtained at each testing site using a GE Logiq Book XP scanner (GE, Waukesha, Wisconsin) with a 38 mm linear array transducer (10 MHz, 8L-RS) and an imaging depth of 40 mm. The focal depth was kept at 12.5 mm. Ultrasound images were obtained longitudinal to the long axis of the extremity and parallel to the orientation of the four-electrode arrangement. The thickness of the subcutaneous tissue for each segment were measured and recorded.

After impedance measurements were completed at each body site, multiple ultrasound images were recorded. The penetration sites of the gold-plated needles were commonly visible on the skin after the needles were removed and thus served as useful markers for where impedance measures were performed. Longitudinal and transverse images were obtained over each segment (meridian and control). Video scans were also obtained with the probe oriented longitudinally and moving laterally over both segments and with the probe oriented transversely and moving distally over each segment. The videos were recorded over six seconds while the probe was moved at a rate of 1 cm/sec.

**Figure 3:**
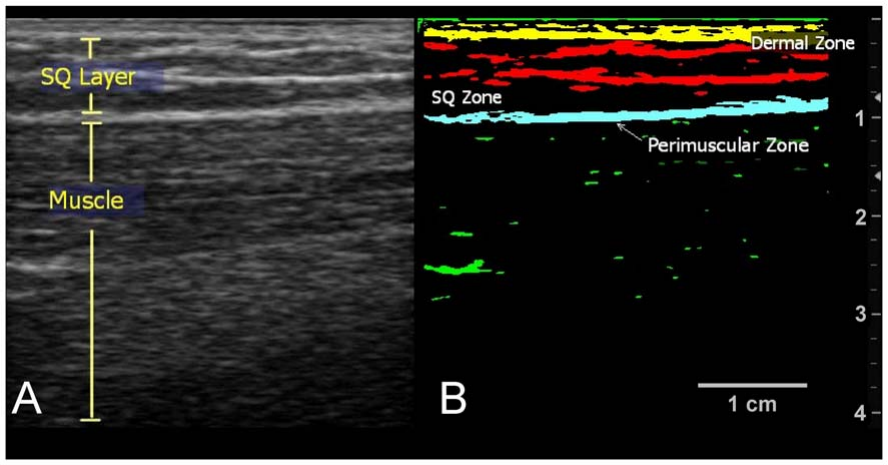
Delineation of Tissue Layers and Zones. (**3A.**) Ultrasound image. This longitudinal image of the thigh shows the subcutaneous and muscle layers. Echogenic bands (white bands) are seen in the subcutaneous layer. (**3B.**) Thresholded image. Thresholding the image delineates the echogenic bands in the dermal, SQ, and perimuscular zones.

The ultrasound images were subsequently analyzed with SigmaScan Pro 5 image analysis software (Systat Software, Chicago, IL). For the longitudinal images acquired over tested segments, the amount of echogenic bands/densities was determined at three separate layers: dermal, subcutaneous, and perimuscular zones (see Figure 3). The echogenic areas were extracted using a threshold filter of 100 out of a maximal pixel intensity of 255 and subsequently quantified by total area (dermal/perimuscular zone) and by area density (subcutaneous zone). The echogenic bands/areas served as surrogates for collageneous bands since past studies have demonstrated that collagen is hyperechogenic and that these echogenic bands corresponded with histological localization of collagen [25], [26]. To account for slight misplacements of the ultrasound transducer over tested segments, three adjacent images both lateral and medial to the perceived segment image and consecutively spaced 1.6 mm apart were also analyzed and averaged to derive the echogenic content at each segment.

In addition, blood vessels within the subcutaneous layer and dermis, running longitudinally, sized greater than 8 mm in diameter, and within 3 cm of the tested segments were identified and recorded.

### E. Impedance Analyses

The impedance measurements performed at each site in duplicate were averaged. The average impedance error between the two measurements was 3.3 Ω. The frequency sweep of impedance under the four needle electrode arrangement did not resemble the typical dielectric dispersion seen with biological tissue and larger current delivering electrodes. The use of needles as current delivering electrodes generated a local polarization effect that lent to a relatively flat frequency response curve and reduced impedances at lower frequencies. Furthermore, the IEC-601 compliant biomedical inputs of the 1294 contributed to additional distortions to the phase readings. For the level of impedance we recorded, the 1294 was accurate within 1% range only at the 10 kHz to 100 kHz frequencies based on the manufacturers' specifications. For these reasons, only the absolute magnitude of the impedance at 10 and 100 kHz was used in the statistical analyses.

### F. Statistical Analyses

To determine whether impedance at acupuncture meridians were different than adjacent controls and to identify factors associated with electrical impedance, a mixed effects model was used to account for across-subject and within-subject factors. The mixed effects model was considered ideal for the clustered, hierarchical data – i.e. within each individual, there are three evaluated body sites (arm, thigh, and calf) and within each body site there are two channels (meridian vs. control). Electrical impedance at 10 kHz and 100 kHz was used as a dependent variable. For our first aim, channel segment (meridian vs. control) and location (arm, thigh, and calf) were repeated factors. If there was evidence of an interaction between the two factors (i.e. segment differences were determined to be location dependent), simple effects within each location were examined based on an F-test corresponding to the appropriate contrast. Simple, compound symmetry, and heterogeneous compound symmetry covariance matrices were tested, and the approach with the lowest AIC (Akaike Information Criterion) value was used (heterogeneous compound symmetry).

For the second aim, two approaches were taken. To identify factors associated with impedance *within sites*, differences in impedance between meridian and control segments were dependent variables while differences in local within-subject factors were used as independent variables (dermal echogenicity, subcutaneous echogenic density, perimuscular echogenicity, presence of vessel, and, subcutaneous thickness). Only variables that were separately measured at the two segments were used as independent variables in this analysis. In cases where the relationship between dependent and independent variables were not linear, logarithmic or powered (e.g. squared) dependent variables were used based on greatest F-test values. To identify factors associated with impedance *between sites*, total impedance served as dependent variables, and between-subject factors (age, gender) and within-subject factors (presence of vessel, dermal echogenicity, subcutaneous echogenic density, perimuscular echogenicity, penetration depth, subcutaneous thickness, and channel site) served as independent variables. Both univariable and multivariable analyses were performed. For multivariable analyses, body site and penetration depth remained a fixed variable for all analyses. A forward stepwise selection approach was used to identify significant associations. Univariable relationships with greatest F-values were inserted and maintained if significant (p>0.10) in the multivariable model. Variables were inserted until the variables were insignificant or AIC (Akaike Information Criterion) values decreased. A heterogeneous compound symmetry covariance matrix was used. Statistical analyses were performed using SAS statistical software (SAS Version 9.2, SAS Institute, Cary, NC).

## Results

Across all subjects and test sites, the mean impedances (± standard deviation) of skin at 10 kHz and 100 kHz, were 352±79 Ω and 446±127 Ω, respectively. Significant variance of impedance existed within subjects as well as between subjects. Within-subject variance of impedance was nearly equal in magnitude to between-subject variance (for 10 kHz, 2,937 and 3,408, respectively; for 100 kHz, 7,289 and 8,999, respectively) and was predominantly attributed to differences in impedance across body sites (thigh vs. arm vs. calf) and not between channels (meridian vs. control).

Compared to its adjacent control, the LI meridian was associated with significant decrease in impedance compared to its control for both 10 and 100 kHz electrical frequencies (Table 1). The Liver and Bladder meridians showed non-significant decreases compared to their respective controls. As a single factor across all three body locations, channel segment (meridian vs. control) was significantly associated with impedance at both 10 kHz and 100 kHz. This statistical significance was attributed to the mixed effects model approach where the reduced mean impedances at the meridians observed across all body sites were taken into account.

**Table 1:** Differences in Electrical Impedance between Meridian and Control.

Segments (N = 28)	10 kHz Impedance		100 kHz Impedance	
	Mean ± SE (Ω)	p-value	Mean ± SE (Ω)	p-value
Large Intestine	345±15		432±23	
LI-Control	355±15		449±23	
Difference (LI – Cont)†	−9.5±3.9	***0.021***	−17.5±6.8	***0.017***
Liver	378±18		490±27	
LV-Control	381±17		503±27	
Difference (Liv – Cont)†	−3.0±9.1	0.74	−12.8±12.8	0.33
Bladder	328±15		398±25	
BL-Control	332±16		407±28	
Difference (BL – Cont)†	−3.9±8.3	0.64	−9.3±12.7	0.47
Overall Channels		***0.03***		***0.007***

† Impedance differences were calculated for each individual at each site. Standard error here represents the variance in impedance for each individual site (within subject), whereas the standard error for the impedances at the meridians and controls represent variance across study subjects (between subject).

To identify anatomical factors that may account for these local *within site* differences, differences in channel impedances between adjacent segments were compared to differences in ultrasound-derived anatomical measures. Table 2 shows the bivariable relationships. At both frequencies, differences in SQ echogenic density and the square root of SQ echogenic density were significantly associated with differences in electrical impedances: segments with greater densities of SQ echogenicity as compared to its adjacent segment had lower impedances. The amount of PM echogenicity had a reverse relationship: segments with greater PM echogenicity had greater impedances. These associations remained significant in multivariable models. SQ thickness, dermal echogenic area, and presence of blood vessels did not demonstrate significant relationships.

**Table 2:** Within-Site Bivariable Analyses: Anatomical Factors associated with Differences in Impedance between Meridian and Control.

Variable	10 kHz Impedance		100 kHz Impedance	
[Control – Meridian]	[Control – Meridian]		[Control – Meridian]	
	Beta (Ω/unit)	p-value	Beta (Ω/unit)	p-value
SQ Thickness (cm)	0.9	0.62	2.1	0.36
Dermal Echogenic Area (cm^2^/frame)	−16.1	0.82	−12.7	0.91
SQ Echogenicity (%)	−***1.08***	***0.02***	−***1.8***	***0.03***
Sqrt (SQ Echogenity)	−***12.0***	***0.006***	−***20.6***	***0.004***
PM Echogenic Area (cm^2^/frame)	46.8	0.09	65.0	0.08

To identify factors associated with impedance *across test sites*, electrical impedance was evaluated as a function of multiple between-subject and within-subject factors. The multivariable relationships are summarized in Table 3. At both frequencies, male gender, increased needle penetration depth, decreased SQ thickness, increased dermal echogenicity and SQ echogenicity were significantly associated with reduced electrical impedance. Furthermore, impedances at the calf and arm were significantly decreased compared to impedances at the thigh. Channel site was not significant in either model. Of all the factors, SQ echogenicity and SQ layer thickness had the greatest associations with impedance as determined by F-values.

**Table 3:** Between-Site Multivariable Analyses: Factors Associated with Electrical Impedance across Body Sites.

Variable	10 kHz Impedance		100 kHz Impedance	
	Beta (Ω/unit)	p-value	Beta (Ω/unit)	p-value
**Between-Subject Effects**				
Gender (ref = Male)	57.67	0.002	92.79	0.01
**Within-Subject Effects**				
Body Site				
Thigh (ref)	*-*	*-*	*-*	*-*
Arm	−31.55	0.006	−52.66	0.003
Calf	−47.63	0.0006	−88.88	<0.0001
Needle Penet Depth (cm)	−110.23	<0.0001	−177.16	<0.0001
Subcutaneous Layer Thickness (cm)	3.3	0.01	4.25	0.05
SQ Echogenic Density (%)	−1.06	0.008	−1.99	0.002

**Figure 4:**
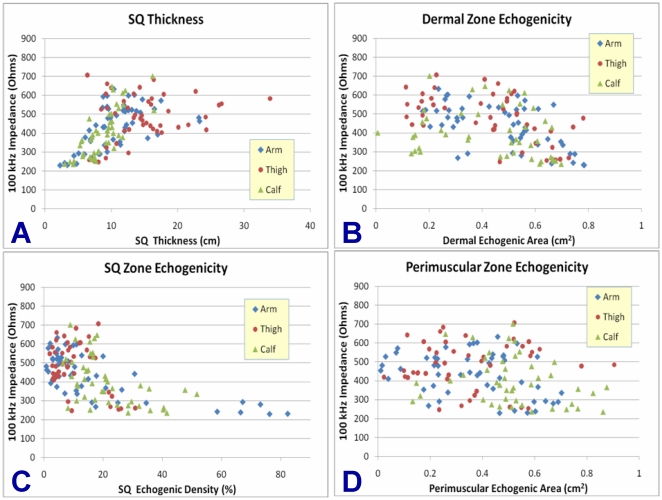
Bivariable Relationships between Ultrasound-Derived Parameters and Impedance. (**4A.**) Bivariable graphical plot: Impedance (100 kHz) vs. SQ Thickness. (**4B.**) Impedance (100 kHz) vs. Dermal Echogenic Area. (**4C**). Impedance (100 kHz) vs. SQ Zone Echogenic Density. (**4D.**) Impedance (100 kHz) vs. Perimuscular Echogenic Area.

The bivariable relationships between ultrasound-derived parameters and 100 kHz impedances are illustrated by body site in Figures 4A–D. The inverse relationship between SQ echogenicity and impedance is evident in Figure 4C and appears to apply to all body sites. Echogenic density greater than 23% is invariably associated with reduced impedance, although high echogenic density is not a prerequisite for lower impedance. For the cases where low impedance was observed at lower echogenicity, sub-analyses did not identify a variable that could account for the lower impedance. SQ layer thickness also demonstrated a correlative relationship with impedance (Figure 4A), while dermal echogenicity showed a less robust correlation (Figure 4B) and perimuscular echogenicity showed no correlation at all (Figure 4D).

## Discussion

In summary, we found an overall trend towards reduced impedance at all meridian segments compared to adjacent controls at 10 and 100 kHz frequency, although this decrease was statistically significant only at the LI meridian segment. The differences in impedances between channel segments were partially attributable to SQ echogenic density. Greater SQ echogenic densities were associated with reduced impedances in both within-site and between-site analyses. This relationship remained significant in multivariable analyses which also accounted for gender, penetration depth, SQ layer thickness, and other ultrasound-derived measures.

Compared to our previous study, this study focused on the subcutaneous tissue at depths of 1–2 cm and not the intermuscular connective tissue. The tight spacing, oblique depth angles, curved longitudinal trajectory, and depth of the intermuscular fascia presented a major challenge to the precise placement of needles in the intermuscular spaces. Furthermore, the intermuscular planes did not consistently coincide with the location of meridians as determined by the acupuncturists. For these reasons, the meridian segments in this study were located by the acupuncturists, while ultrasound images were used as objective markers for any underlying physical structures that may account for differences in electrical impedance.

In addition, compared to the previous study, this study used a widely accepted impedance gain-phase analyzer – Solartron 1260 – and a compatible biological interface - Solatron 1294 – to obtain impedances across multiple frequencies. The low electrical impedances associated with the uninsulated needles, however, limited the accurate range (based on manufacturer specifications) of the Solartron 1294 biological interface to between 10 and 100 kHz. This differs with the 3.3 kHz frequency used in the original study.

Despite these differences, our study identified lower impedances at the LI meridian segment compared to its adjacent control at both frequencies. Mean impedances at the other two meridian sites showed a trend toward reduced levels for both frequencies although neither reached statistical significance. This result was similar to the findings identified at the Pericardium meridian (significantly reduced impedance) and Spleen meridian (no significant difference) in our previous study. Why this significant relationship occurred at the LI and PC meridian site and not the others is unclear. It is possible that the 1.3 cm separation distance between meridian and control segments was insufficient for the lower extremities.

Subcutaneous echogenicity was significantly associated with impedance in within-site analyses. In between-site analyses, electrical impedance was invariably below 450 Ohms for SQ echogenic densities above 23%. This inverse association between SQ echogenicity and impedance remained significant even after accounting for SQ thickness, gender, presence of large vessels and other potential confounders. Furthermore, this relationship existed at all tested body sites, strongly suggesting the generalizability of this association to other body areas. Additionally, this relationship may not be restricted to the SQ layer alone. Bivariable analyses revealed that dermal echogenicity was also inversely related with impedance. Meanwhile, the insignificant association between PM zone echogenicity and electrical impedance may be attributed to the insertion of needle electrodes to the depths of SQ thickness. In such cases, the needle did not frequently penetrate the PM layer and it is theoretically possible for the impedances to be registered at higher levels when electrical currents are shunted away from the inner voltage sensing electrodes towards deeper PM layer (thus explaining the higher impedances seen with greater PM echogenicity seen in Table 2).

Based on biophysical and histological studies, these echogenic bands/areas are highly correlated with the location of collagen fibers and more specifically the bands of coalesced collagen fiber. [25], [26]. Consequently, our results suggest that collagen may be the macromolecule responsible for the observed lower impedances. Other studies support the evidence that collagen is characterized by low electrical impedance. A recent MREIT (Magnetic Resonance Electrical Impedance Tomography) study, for instance, performed conductivity imaging of the lower extremity in four healthy volunteers and observed significantly greater conductivities in the crural fascia and intermuscular septum (both areas known to contain large amounts of collagen) compared to muscle [27]. The authors, however, ascribed this observation to presence of “conductive fluid in those regions”. Another study measured electrical impedance in irradiated and normal muscles in rat hind legs and found reduced impedances in irradiated legs proportional to the amount of radiation administered. The authors concluded that the “presence of interstitial fibroplasia [as a result of irradiation] may be providing a short around the muscle tissue, reducing the intracellular structural effects of muscle‥”, although direct, quantitative evidence for this hypotheses was not given [28]. A third study evaluated the electrical impedance of normal and chronically infarcted left ventricular myocardium from sheep and reported a highly significant correlation between impedance and collagen content based on histological analyses [29].

From a theoretical standpoint, other authors have conjectured that the highly ordered, crystalline arrangement of collagen would confer it with various semiconductive properties, although no direct, objective data were provided in such cases [30], [31], [32]. To our knowledge, this study is the first to demonstrate a statistically significant association between subcutaneous collagenous bands and electrical impedance *in vivo*. The ultrasound images provided the means to not only quantify SQ collagenous bands but also account for other subcutaneous structures that may influence impedance measures and thus potentially confound the observed relationship. If the low impedance property of collagen and collagenous bands is further corroborated, the use of impedance-based devices for diagnostic purposes (such as BIA) would have an additional biological foundation particularly for conditions where the collagen and extracellular matrix are altered. This may include scarring from radiation or injury, inherited collagen disorders, or cancers where distortions of extracellular matrix by malignant cells are increasingly being recognized [33], [34], [35].

From the perspective of acupuncture, this study suggests that SQ collagenous bands may underlie the reduced impedance described at acupuncture meridians. Interestingly, SQ echogenicity was the only recorded variable associated with impedance in both within-site and between-site analyses. In other words, this association was not only generalizable to multiple body sites but also specific enough to account for local differences. Nevertheless, the physiological significance of this relationship remains unclear. The magnitude of differences between channel segments was small and unlikely to be physiologically significant. The use of uninsulated needles may partly explain this small difference since the shaft acts to short-circuit all the contacted tissue and thus average across multiple layers. The four electrode arrangement may also attenuate significant differences between channels, especially if currents are shunted away from current sensing electrodes towards an adjacent low-impedance pathway or if the segments between the current and measuring electrodes have high impedances (zone of negative sensitivity) [36]. A more focused, depth-specific approach using insulated needles will be needed to appropriately evaluate the impedance of collagen fibers and to investigate its physiological importance, if any exists.

There are additional limitations to this study. First, due to added technical distortions stemming from the IEC-601 compliant biomedical inputs (Solartron 1294), information on impedance phase was essentially lost. Such information would greatly clarify how the meridians differed and how collagenous bands are associated with reduced impedance. Second, the needle electrodes were not inserted in muscle. The effects of perimuscular fascia and muscle were not directly assessed. It is possible that these deeper layers also play a role in local electrical impedance. Third, the meridian segments were located by acupuncturists and not by an objective standard. As a result, the reported differences (or lack thereof) in impedance between channel segments are subject to bias. Finally, the study is limited by small sample size. A larger study sample may have generated more robust results that can be interpreted with better confidence.

Despite these limitations, this study adds substantial insights into the electrical impedance associated with acupuncture meridians and connective tissue. Collagenous bands, represented by increased ultrasound echogenicity, are significantly associated with lower electrical impedance and may be the common denominating factor for the reduced impedances reported at certain acupuncture meridians at both subcutaneous and intermuscular depths. If so, the SQ collagenous bands may present an objective basis for the hitherto elusive acupuncture meridians. Furthermore, the study's findings may provide important insights into the nature of acupuncture-related treatments and into the relevance of collagen in bioelectrical measurements.
